# Effect of Microstructure on Photo-Induced Current Characteristics of Eu^2+^-Doped SrAl_2_O_4_

**DOI:** 10.3390/ma15186254

**Published:** 2022-09-08

**Authors:** Hyunseok Lee, Seung-Woo Lee

**Affiliations:** Department of Fine Chemistry, Seoul National University of Science and Technology, 232 Gongneung-ro, Nowon-gu, Seoul 01811, Korea

**Keywords:** Eu^2+^-doped SrAl_2_O_4_, photocurrent, microstructure, grain boundary

## Abstract

Research interest is active in Eu^2+^/Dy^3^-doped SrAl_2_O_4_ phosphors, and the photocurrent characteristics of Eu^2+^-doped SrAl_2_O_4_ without Dy^3+^ was investigated extensively. However, it exhibits a low photocurrent of ~1 μA owing to high resistance. In this study, the changes in a photo-induced current with grain size were examined. The effect of the processing methods on the microstructure and photocurrent characteristics of Eu^2+^-doped SrAl_2_O_4_ phosphors was investigated. (Sr_0.99_Eu_0.01_)Al_2_O_4_ powders were synthesized using a conventional solid-state method and calcined at 1350 °C under a 3% H_2_/(Ar + H_2_) reducing gas atmosphere. Sample pellets were fabricated as conventional and hot-press-sintered bodies at 1400 °C. A thin film of the sample was deposited on an alumina substrate by radio-frequency magnetron sputtering. Scanning electron microscopy revealed that the hot-press-sintered body has larger grains than the conventionally sintered body. The photo-induced current of the hot-press-sintered body at 400 lx was ~100 and ~1000 times higher than those of the conventionally sintered body and thin film, respectively. Impedance analysis confirmed that this dramatic increase in the photo-induced current is closely related to the increase in the grain size and crystallinity of the sample. This study verifies the applicability of Eu^2+^-doped SrAl_2_O_4_ as both a phosphor and photosensor.

## 1. Introduction

Advancements in science and technology have led to demands for the development and application of new materials with improved properties and performances. To meet these demands, research on ceramic materials has been actively conducted, from nanoparticles to various sintering methods, both in the industry and academia. In this context, phosphorescent phosphors, which absorb incident light energy and emit light in the dark, have attracted much attention, and Eu^2+^-doped SrAl_2_O_4_ is one of the promising phosphorescent phosphors [1,2,3,4].

Eu^2+^-doped alkaline earth aluminates, which are complex metallic oxides represented by MAl_2_O_4_:Eu^2+^ (M = Sr, Ca, Ba, Mg, etc.), are long-lasting phosphorescent materials [5,6,7,8,9]. These long-afterglow phosphors slowly release optical energy accumulated with prior exposure to sunlight or fluorescent light; the optical energy is released in the form of light emission. In 1996, Murayama et al. reported a remarkable improvement in afterglow characteristics by adding Dy to the existing SrAl_2_O_4_:Eu^2+^ composition as a co-activator [10]. This material exhibits enhanced afterglow properties without the addition of tritium (H-3) or promethium (Pm-147), which are radioactive isotopes used to improve afterglow properties. In addition, most phosphors use sulfide compounds such as Cu-doped ZnS [11,12]. However, the sulfide-based compound is affected by moisture and carbon dioxide, thereby leading to the degradation of phosphorescence properties, as well as environmental pollution [13,14]. In 1971, Abbruscato measured the photocurrent flowing over the SrAl_2_O_4_:Eu^2+^ phosphor by positioning the phosphor between quartz and an Au layer and applying a DC voltage of 400 V to both ends [15]. The author argued that the holes were the carriers contributing to the afterglow properties and optical rectification of Eu^2+^-doped SrAl_2_O_4_. To support this argument, the photoluminescence and photo-induced current were measured to confirm that the afterglow properties, and optical rectification showed the same pattern of reduction.

**Figure 1:**
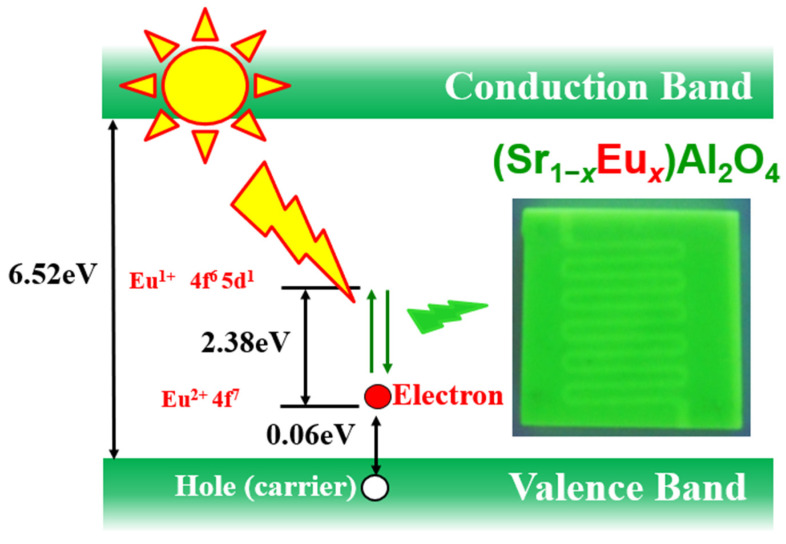
Schematic of the photocurrent generation mechanism of Eu^2+^-doped SrAl_2_O_4_.

Figure 1 shows the mechanism of the optical rectification of Eu^2+^-doped SrAl_2_O_4_. SrAl_2_O_4_ is an insulator with a band gap of 6.52 eV. However, Eu^2+^ doping creates an acceptor level that is 0.06 eV above the valence band. In the absence of light, electrons and holes form a pair such that no current flows. However, with incident light at a wavelength less than 500 nm, Eu^2+^ electrons at the acceptor level are excited due to the 4f^7^ to 4f^6^5d^1^ transition (Figure 1), and current flows with holes as carriers. Based on this theory, studies have been conducted on the photocurrent of a thick film of Eu^2+^-doped SrAl_2_O_4_ slurry and its sintered body [10,16,17,18]. ZnO-SrAl_2_O_4_:Eu (ZnO-SAO:Eu) nanocomposites have been reported as materials suitable for applications in optical fiber thermometers by Ghahrizjani et al. [19]. Moreover, Nazarov et al. [20] reported the luminescence mechanism by calculations of the crystal field splitting of the Eu^2+^ 5d states. The results of these previous studies suggest that the particle size and microstructure have a significant effect on the photoluminescence of Eu^2+^-doped SrAl_2_O_4_ with Dy^3+^ as the co-dopant.

In this study, we demonstrated the photocurrent characteristics of Eu^2+^-doped SrAl_2_O_4_ for the first time by controlling its microstructure. Furthermore, the grain size of Eu^2+^-doped SrAl_2_O_4_ was controlled by producing sintered bodies using the conventional method and hot pressing, in addition to thin-film samples. Additionally, we confirmed the internal resistance of the grain boundary by impedance analysis.

## 2. Materials and Methods

### 2.1. Synthesis of Eu^2+^-Doped SrAl_2_O_4_ Powder

SrCO_3_ (Kojundo, 99.9% purity) was used as a precursor for SrAl_2_O_4_ powder before fabricating the SrAl_2_O_4_-sintered bodies and thin films. Al_2_O_3_ (Kojundo, 99.99% purity) was used as the Al source. To this, high-purity B_2_O_3_ (Kojundo, 99.9% purity) as flux and Eu_2_O_3_ (Uranus Chemicals, 99.99% purity) as an activator were added to synthesize (Sr_1-*x*_Eu*_x_*)Al_2_O_4_ powder [21].

The synthesis procedure for the powder was as follows. The stoichiometric ratio of Sr:Eu:Al of the (Sr_1-*x*_Eu*_x_*)Al_2_O_4_ specimen, i.e., 0.99:0.01:1, was weighed and mixed for 8 h in a wet process using ethanol. The mixed specimens were dehydrated using a vacuum drier (SB-651, EYELA, Tokyo, Japan). Mixed specimens were synthesized using an atmosphere tube furnace, and a synthesis temperature of 1350 °C was selected after preliminary experiments. The (Sr_0.99_Eu_0.01_)Al_2_O_4_ specimen obtained by calcination was ground using a mortar and passed through 100-mesh sieves. The sieved specimens were pulverized using a planetary mill (PM400, Retsch, Haan, Germany) to fabricate high-density sintered bodies and targets. The pulverization was a dry process for 30 min at 200 rpm, and the powder was sieved through a 120-mesh sieve to obtain a uniform particle size of the ground specimen.

### 2.2. Fabrication of Sintered Bodies

The conventionally sintered body sample was subjected to a pressure of 1200 kgf/cm^2^ after the specimen was placed in a mold. The formed specimen was sintered at 1400 °C. The specimen was reduced and sintered while injecting 3% H_2_/(Ar + H_2_) reducing gas into the tube furnace at a rate of 800 cc/min. The samples were heat-processed for 2 h at a sintering temperature of 1400 °C and heating rate of 5 °C/min.

Another specimen was fabricated using hot-press sintering. In the hot-press sintering process, (Sr_0.99_Eu_0.01_)Al_2_O_4_ powder was charged into a graphite mold using a hot press (KHP-200, Kovaco, Inchon, Korea). To minimize the reaction between the powder and graphite mold, a graphite sheet was placed on the bottom and side of the mold, and a boron-nitride spray was applied to the upper punch. The specimen was reduced and sintered under a 3% H_2_/(Ar + H_2_) reducing gas atmosphere injected at a rate of 800 cc/min. The sintering temperature was 1400 °C, which was similar to that employed to prepare the conventionally sintered body sample, and four samples were produced through heat processing for 30 min, 2 h, 4 h, and 6 h, and hot pressing at a pressure of 200 kgf/cm^2^. However, the sample sintered for 6 h could not be characterized because it was destroyed under high-temperature and high-pressure conditions, as shown in Figure S1. The surfaces and contours of all sintered body samples were ground with SiC sandpaper, following which the samples were processed with ultrasonic cleaning using isopropyl alcohol and dried at 80 °C.

### 2.3. Fabrication of Eu^2+^-Doped SrAl_2_O_4_ Thin Film

A single-phase target of the fabricated (Sr_0.99_Eu_0.01_)Al_2_O_4_ composition was deposited on an alumina substrate (Al_2_O_3_, RNDKorea, Seoul, Korea) using radio frequency (RF) magnetron sputtering. An experiment was conducted to examine the optimal processing conditions before fabricating a film with the Sr-substituted doping target, following which a thin film was fabricated using the optimized processing conditions. The substrate had a size of 1 cm × 1 cm and was supersonically cleaned using acetone for 15 min, methanol for 15 min, and deionized water for 15 min and then dehydrated using N_2_ gas before film deposition. The pressure before the deposition was less than 6 × 10^−4^ Pa, and a thin film was deposited at a pressure of 2.6 Pa. The (Sr_0.99_Eu_0.01_)Al_2_O_4_ target was sputtered at 3 W/cm^2^. The thin film was fabricated at a fixed substrate temperature of 400 °C and heat-processed under a reducing atmosphere formed by injecting 3% H_2_/(Ar + H_2_) reducing gas at a rate of 800 cc/min. The heat processing temperature, duration, and temperature ramp rate were 1000 °C, 2 h, and 5 °C/min, respectively.

### 2.4. Fabrication of Top-Electrode Thin Film

The top electrode of each sample was formed by depositing indium tin oxide (ITO) thin films using RF magnetron sputtering. An interdigitated patterned shadow mask was used to form the electrode pattern.

### 2.5. Characterization of the Samples

The morphology of each sample was measured and confirmed using scanning electron microscopy (SEM, Inspect F50, FEI, Lausanne, Switzerland). The crystallization of all samples was investigated by X-ray diffraction (XRD, D MAX2200, Rigaku, Tokyo, Japan, Cu Kα, 40 kV/30 mA, and wavelength of 1.54056 Å). The photocurrent was measured by applying a DC voltage of 220 V while controlling the light intensity using a solar simulator (66984, Newport, Irvine, CA, USA) (237, Keithley, Cleveland, OH, USA).

## 3. Results and Discussion

**Figure 2:**
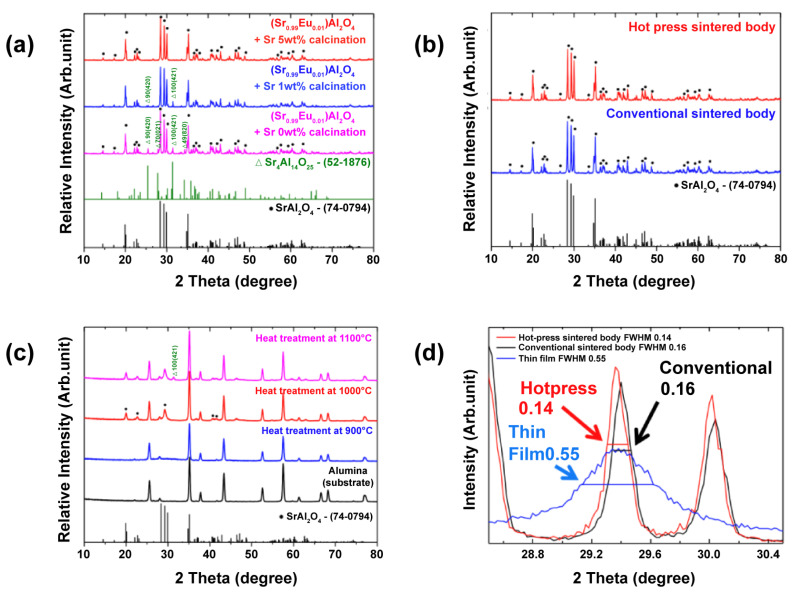
(**a**) X-ray diffraction (XRD) patterns of (Sr_0.99_Eu_0.01_)Al_2_O_4_ powder calcined at 1350 °C. (**b**) XRD patterns of a (Sr_0.99_Eu_0.01_)Al_2_O_4_-sintered body at 1400 °C. (**c**) XRD patterns of (Sr_0.99_Eu_0.01_)Al_2_O_4_ thin films deposited on alumina substrates and annealed at 900 °C, 1000 °C, and 1100 °C. (**d**) The full width at half maximum (FWHM) of (Sr_0.99_Eu_0.01_)Al_2_O_4_ samples obtained from the XRD patterns (2θ = 29.4°).

Figure 2a shows the XRD pattern of (Sr_0.99_Eu_0.01_)Al_2_O_4_ powder calcined for 2 h at 1350 °C. The (Sr_0.99_Eu_0.01_)Al_2_O_4_ powder was added according to the chemical ratio in the quantitative relationship, and the synthesized powder had the second phase of Sr_4_Al_14_O_25_ (JCPDS No. 52-1876) (2θ = 25.5, 31.5°). The use of an alumina boat to synthesize the (Sr_0.99_Eu_0.01_)Al_2_O_4_ powder was judged to be the cause of this second phase because the second phase, which lacks Al_2_O_3_, was formed through the reaction with Al_2_O_3_ that constitutes the alumina boat. Therefore, to obtain single-phase (Sr_0.99_Eu_0.01_)Al_2_O_4_ powder, excess SrCO_3_ was added for synthesis. However, the second phase was still present in the powder with the addition of 1 wt% SrCO_3_. Hence, 5 wt% excess SrCO_3_ was added to the powder, and single-phase SrAl_2_O_4_ (JCPDS No. 74-0794) was finally obtained. 

Figure 2b shows the XRD patterns of the sintered pellets. The obtained (Sr_0.99_Eu_0.01_)Al_2_O_4_ powder was sintered for 30 min, 2 h, 4 h, and 6 h at 1400 °C, and its phases were analyzed using XRD. The pellets showed the same XRD pattern as the powder, as shown in Figure 2b. For the conventionally sintered body sample, an alumina boat was used for sintering in the same manner as described above. However, a single-phase sample was obtained by spreading the (Sr_0.99_Eu_0.01_)Al_2_O_4_ powder under the molded sample to prevent contamination. The hot-press-sintered body was prevented from contamination using a carbon mold and boron-nitride coating, thereby obtaining a single-phase sample. Figure 2c shows the XRD results of the (Sr_0.99_Eu_0.01_)Al_2_O_4_ thin films. The (Sr_0.99_Eu_0.01_)Al_2_O_4_ thin-film sample annealed at 900 °C exhibited an amorphous phase, and only the alumina peak of the substrate was observed. For the (Sr_0.99_Eu_0.01_)Al_2_O_4_ thin-film sample annealed at 1000 °C, the main peaks of SrAl_2_O_4_ were observed. However, in the case of the (Sr_0.99_Eu_0.01_)Al_2_O_4_ thin-film sample annealed at 1100 °C, the second phase, Sr_4_Al_14_O_25_, was observed. Therefore, post-annealing at 1000 °C was confirmed to be the optimal condition for the samples. Figure 2d shows the full width at half maximum (FWHM) obtained from the XRD patterns (2θ = 29.4°). The conventionally sintered body, hot-press-sintered body, and thin-film sample sintered at 1400 °C for 2 h were measured. Peaks between 28.2° and 30.2°, i.e., the main peaks, were observed. The FWHM values were 0.16, 0.14, and 1.55 for the conventionally sintered body, hot-press-sintered body, and thin film, respectively. Therefore, the results confirmed that the hot-press-sintered body sample exhibited the highest level of crystallization, while the thin film showed the lowest level of crystallization.

**Figure 3:**
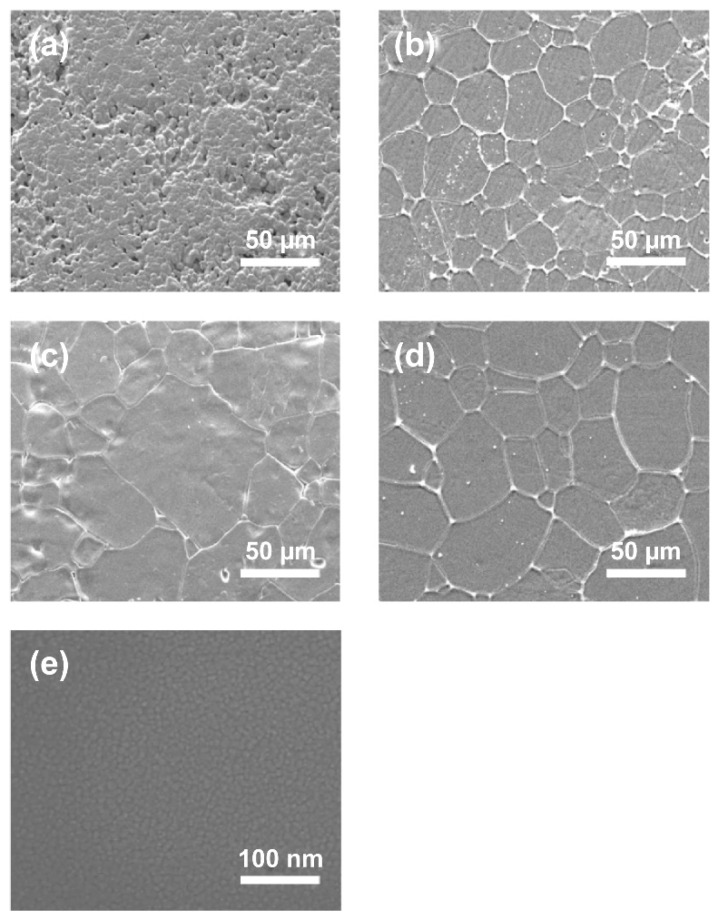
SEM images of (Sr_0.99_Eu_0.01_)Al_2_O_4_-sintered body: (**a**) conventionally sintered body; (**b**,**c**), and (**d**) hot-press-sintered body that were heat processed for 30 min, 2 h, and 4 h, respectively. (**e**) SEM images of the (Sr_0.99_Eu_0.01_)Al_2_O_4_ thin film.

Figure 3 shows SEM images of the (Sr_0.99_Eu_0.01_)Al_2_O_4_ pellet samples and thin films. The conventionally sintered body was found to consist of several micro-sized particles, whereas that obtained with hot pressing showed particle growth from several tens of micrometers to 100 μm [22,23]. In addition, in a hot-press-sintered body, pressure is applied during sintering, resulting in a sintered body with a dense structure. The relative density of the conventionally sintered body was 76.9%, while that of the hot-press-sintered body was 99.3%, almost reaching the theoretical density obtained using a gas pycnometer (Accupyc 1345, Micromeritics, Norcross, GA, USA). Figure 3e shows an SEM image of the surface of the (Sr_0.99_Eu_0.01_)Al_2_O_4_ thin films. Grain sizes of approximately 10 nm or less were observed for the (Sr_0.99_Eu_0.__01_)Al_2_O_4_ thin films. In addition, the formation of a film with a dense structure was verified.

For a more detailed analysis of the grain sizes of the samples, SEM images and the intercept method were used. The line intercept method was developed by Canfield in the 1940s. In this technique, a random straight line is drawn through the SEM image and the number of grain boundaries intersecting this line is counted. The average grain size is obtained by dividing the number of intersections by the actual line length. Table 1 lists the average grain sizes obtained from the SEM images. The conventionally sintered body sample had a grain size of approximately 4.8 μm, while the hot-press-sintered body samples had grain sizes of approximately 35.5 μm, 42.9 μm, and 59.1 μm for soaking times of 30 min, 2 h, and 4 h, respectively. As the soaking time increased, the grain size also increased. A comparison of the results shows that the grain size of the hot-press-sintered body was approximately 10 times larger than that of the conventionally sintered body for the same soaking time. Therefore, the sintering method is an important parameter in terms of the increase in grain size. Compared with the above samples, the thin-film sample was found to have a very small grain size of approximately 10 nm.

**Figure 4:**
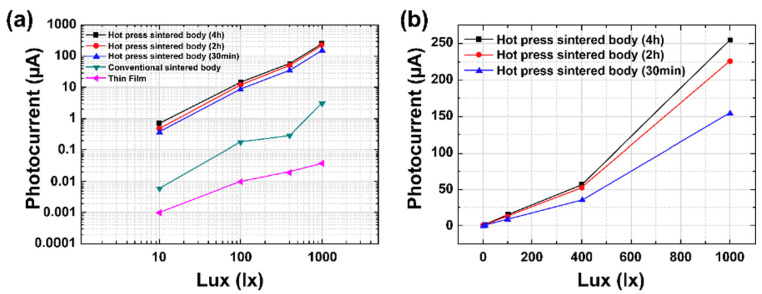
(**a**) Photocurrent of (Sr_0.99_Eu_0.01_)Al_2_O_4_ samples with increasing light intensity. (**b**) Photocurrent of the samples with various soaking times.

Figure 4a shows the changes in photocurrent with the light intensity for different fabrication methods of (Sr_0.99_Eu_0.__01_)Al_2_O_4_. The photocurrent was obtained in proportion to the light intensity in all the specimens. Linearity was also excellent above 400 lx. With regard to the sintering method’s dependence, the photocurrent of the hot-press-sintered body was approximately 250 μA at 1000 lx, which was approximately 100 times higher than that of the conventionally sintered body. The photocurrent of the (Sr_0.99_Eu_0.01_)Al_2_O_4_ thin film was measured as 38 nA at 1000 lux. The examination of the photocurrent characteristics of the (Sr_0.99_Eu_0.01_)Al_2_O_4_ material showed that the photocurrent increased with increasing grain size. This is attributed to the decrease in 3D defects and grain boundaries, which play a role in the resistance inside the ceramic sintered body, due to the improved grain growth and increased density in the hot-press process. Figure 4b shows the measured photocurrent with respect to the soaking time in the hot-press-sintered body samples. The sample with a soaking time of 2 h showed a dramatic 50% increase in the photocurrent and a 20.8% increase in the average grain size in comparison with the 30-min sample. However, the sample with a soaking time of 4 h showed a 66% increase in the photocurrent and a 66% increase in the average grain size in comparison with the 30-min sample.

**Figure 5:**
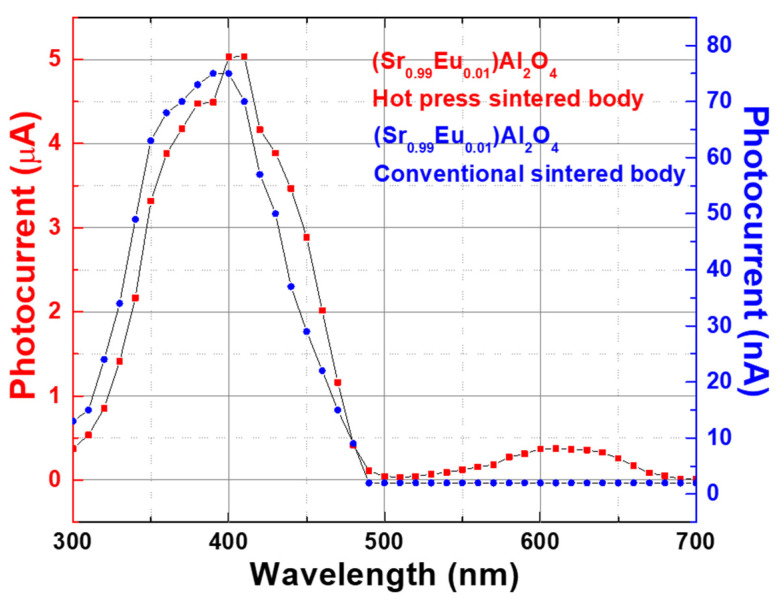
Changes in photocurrent as a function of the wavelength of the conventionally sintered body and hot-press-sintered body samples for 2-h sintering at 1400 °C.

Figure 5 shows the changes in the photocurrent with the wavelength of the sample for different fabrication methods of (Sr_0.99_Eu_0.__01_)Al_2_O_4_. The photocurrent characteristics of the conventionally sintered body sample and hot-press-sintered body sample were measured. Both samples were sintered at 1400 °C, and the soaking time was 2 h. A 100-fold difference existed in the photocurrent between the conventionally sintered body and hot-press-sintered body samples. However, both samples showed similar patterns in the graph of the photocurrent measurements according to the wavelength. This is attributed to the characteristics of the (Sr_0.99_Eu_0.01_)Al_2_O_4_ material, in which the photocurrent is generated at a wavelength of 300–500 nm. This trend is similar to that reported by Yuan et al. [18] using single-crystal, Eu^2+^-doped SrAl_2_O_4_. The photocurrent mainly occurs in the wavelength range of 300–500 nm, and the highest photocurrent is generated at a wavelength of 400 nm [20]. The photocurrent values obtained according to the wavelength were smaller than those obtained according to the light intensity. This was because of the decrease in light intensity due to the use of a monochromator. As shown in Figure 4a, the (Sr_0.99_Eu_0.01_)Al_2_O_4_ thin films have a low photocurrent of 38 nA at 1000 lx. For the thin films, the use of a monochromator decreased the light intensity to such an extent that photocurrent measurement was not possible.

**Figure 6:**
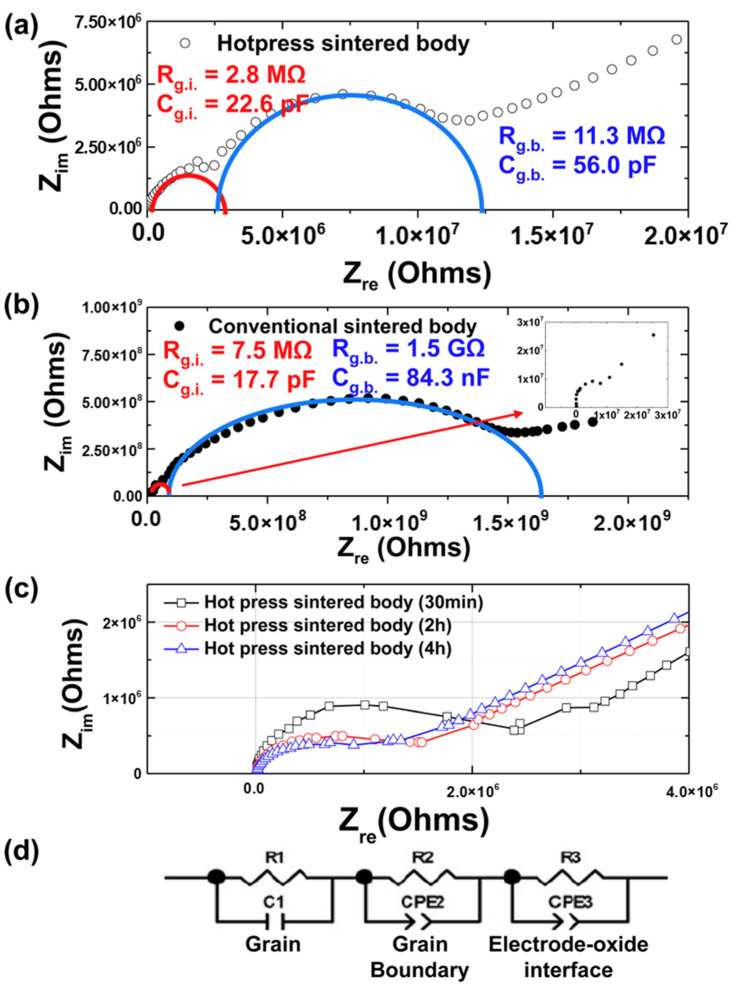
Complex impedance plane of (**a**) the hot-press-sintered body and (**b**) conventionally sintered body. The samples in both (**a**,**b**) were sintered at 1400 °C for 2 h. (**c**) Results of impedance spectroscopy for different soaking times. (**d**) Equivalent circuit model for the sintered body.

Figure 6 shows the impedance spectra of the conventionally and hot-press-sintered bodies. In general, the electrical conductivity of a ceramic material is determined by a combination of the electrical conductivity characteristics at the interface between the specimen and electrode and those at the grain boundaries and interior. The overall resistance and electrical conductivity can be identified by measuring the current flow during the application of a DC voltage to the specimen attached to an electrode. However, this analysis should be performed using AC voltage to identify and understand the resistance at the grain boundaries and interior [21]. In 1969, Bauerle was the first to apply the complex impedance method to electronic ceramics, in which the complex admittance was obtained to analyze the electric conductivity of solid electrolytes of yttria-stabilized zirconia [24]. Impedance analysis can be used to investigate the electrical properties at the grain interior and grain boundaries of ceramics [25,26]. Therefore, we conducted an impedance analysis to compare the resistances at the grain interior and grain boundaries according to the grain sizes by observing the Nyquist plots. Subsequently, this method was extended to several applications [27,28,29].

Figure 6a shows a Nyquist plot of the data obtained from the impedance analysis of the hot-press-sintered body. The resistance and capacitance were calculated based on the equivalent circuit shown in Figure 6d. The semicircle in the high-frequency range represents the characteristics of the grain interior, which showed resistance and capacitance values of R_g.i._ = 2.8 MΩ and C_g.i._ = 22.6 pF, respectively. In addition, the second semicircle yields R_g.b._ = 11.3 MΩ and C_g.b._ = 56 pF, corresponding to the grain boundaries. We calculated the characteristics of the hot-press-sintered body by fitting the Nyquist plot into a simulation using Zplot (Scribner, Southern Pines, NC, USA). The third semicircle was obtained by the polarization of space charge and by non-ohmic contact. Because (Sr_0.99_Eu_0.01_)Al_2_O_4_ is a p-type material, a Pt electrode with a high work function is preferred; however, given that optical transmission declines and light cannot reach the sintered body if a Pt electrode is used, using ITO electrodes with good light transmission seems advantageous. This leads to the formation of a non-ohmic contact, thereby causing the polarization of space charge and resulting in the third semicircle [30,31].

Figure 6b shows a graph of the impedance data for the conventionally sintered body. The semicircle in the high-frequency range shows the characteristics of the grain interior, which exhibited values of R_g.i._ = 7.5 MΩ and C_g.i._ = 17.7 pF. In addition, the second semicircle can be used to obtain values of R_g.b._ = 1.5 GΩ and C_g.b._ = 84.3 nF. The values corresponding to the conventionally sintered body sample were calculated using the same method as that for the hot-press-sintered body sample. Moreover, the tail-shaped plot shows the polarization of space charge, which is similar to that obtained with the hot-press-sintered body. Figure 6c shows the results of impedance spectroscopy for different soaking times. The resistance in the grain interior decreased with an increase in the soaking time.

By comparing the values obtained above, the hot-press-sintered body was found to exhibit a lower resistance at the grain boundary and interior and a higher capacitance than the conventionally sintered body. The resistance at the grain interiors of the sintered body fabricated with hot pressing decreased because its crystallization improved with hot pressing, thereby reducing the crystal defects, as well as decreasing the disappearance of carriers in crystal defects. This reasoning was validated by morphological observations using SEM, the relative density values according to the gas pycnometer, and the FWHM values shown in Figure 2d. In addition, this detrimental outcome proves the expectation that the grain-boundary resistance decreases with a decrease in the grain boundaries due to an increase in the grain size, thereby increasing the photocurrent. Generally, the increase in grain size is related to the increase in photocurrent [32,33,34]. Hwang et al. [35] reported the impact of grain size on optoelectronic performance. As expected, after measuring the photocurrent changes with respect to the light intensity, the grain-boundary resistance of the hot-press-sintered body was found to be lower than that of a conventionally sintered body. However, because the resistance was extremely high, the electrical behavior inside the thin film could not be determined.

## Figures and Tables

**Table 1:** Average grain size of (Sr_0.99_Eu_0.01_)Al_2_O_4_ samples calculated using the intercept method under different fabrication conditions.

Sample Name	GRAIN SIZE
Conventionally sintered body	4.8 ± 0.2 μm
Hot-press-sintered body (30 min)	35.5 ± 3.6 μm
Hot-press-sintered body (2 h)	42.9 ± 4.4 μm
Hot-press-sintered body (4 h)	59.1 ± 4.4 μm
Thin film	~10 nm

## Data Availability

Not applicable.

## Supplementary Materials

The following supporting information can be downloaded at: https://www.mdpi.com/article/10.3390/ma15186254/s1, Figure S1: Photograph of (Sr_0.99_Eu_0.01_)Al_2_O_4_-sintered body: hot-press-sintered body that was heat processed for 6 h.
