# Supplementary material for: Effect of Microstructure on Photo-Induced Current Characteristics of Eu2+-Doped SrAl2O4
Source: Materials (Basel). 2022 Sep 8;15(18):6254. doi: 10.3390/ma15186254 (PMC9501496; doi:10.3390/ma15186254)
Supplement: Supplementary file 1 [file materials-15-06254-s001.zip › materials-1900428-supplementary.pdf]

Supplementary Material

Effect of Microstructure on Photo-Induced Current Characteristics of Eu^{2+}-Doped $SrAl_2O_4$

Hyunseok Lee[1] and Seung-Woo Lee[1,*]

[1] Department of Fine Chemistry, Seoul National University of Science and Technology, 232 Gongneung-ro, Nowon-gu, Seoul 01811, Korea
* Correspondence: swlee@seoultech.ac.kr;

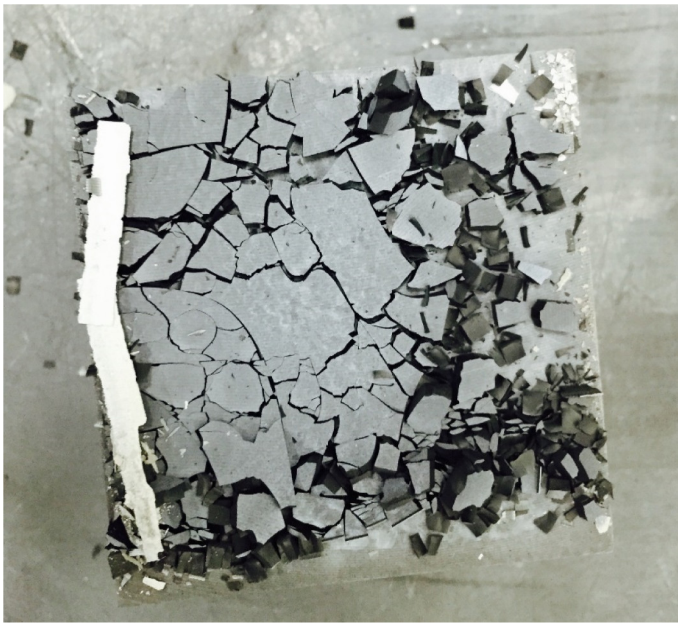

Figure S1. Photograph of $(Sr_{0.99}Eu_{0.01})Al_2O_4$ sintered body: hot-press sintered body that was heat processed for 6 h.
